# Efficacy of 'Tailored Physical Activity’ in reducing sickness absence among health care workers: design of a randomised controlled trial

**DOI:** 10.1186/1471-2458-13-917

**Published:** 2013-10-02

**Authors:** Lotte Nygaard Andersen, Birgit Juul-Kristensen, Kirsten Kaya Roessler, Lene Gram Herborg, Thomas Lund Sørensen, Karen Søgaard

**Affiliations:** 1 Institute of Sports Science and Clinical Biomechanics, Faculty of Health Sciences, University of Southern Denmark, Odense, Denmark; 2 Institute of Occupational Therapy, Physiotherapy and Radiography, Bergen University College, Bergen, Norway; 3 Department of Psychology, Faculty of Health Sciences, University of Southern Denmark, Odense, Denmark; 4 Senior Citizen and Health Department, Social and Health Affairs, Municipality of Sonderborg, Sonderborg, Denmark; 5 Medical Department, Hospital of Southern Jutland, Region of Southern Denmark, Denmark

**Keywords:** 'Tailored Physical Activity’, Health care workers, Musculoskeletal disorders, Prevention, Sickness absence

## Abstract

**Background:**

Health care workers have high physical work demands, involving patient handling and manual work tasks. A strategy for prevention of work-related musculoskeletal disorders can enhance the physical capacity of the health care worker. The aim of this study is to evaluate the efficacy of 'Tailored Physical Activity’ for health care workers in the Sonderborg Municipality.

**Methods/Design:**

This protocol describes the design of a randomised controlled trial to assess the efficacy of 'Tailored Physical Activity’ versus a reference group for health care workers in the Sonderborg Municipality. Inclusion criteria to be fulfilled: health care workers with daily work that includes manual work and with the experience of work-related musculoskeletal pain in the back or upper body.

All participants will receive 'Health Guidance’, a (90-minute) individualised dialogue focusing on improving life style, based on assessments of risk behaviour, on motivation for change and on personal resources. In addition, the experimental groups will receive 'Tailored Physical Activity’ (three 50-minute sessions per week over 10 weeks). The reference group will receive only 'Health Guidance’.

The primary outcome measure is the participants’ self-reported sickness absence during the last three months due to musculoskeletal troubles, measured 3 and 12 months after baseline.

In addition, secondary outcomes include anthropometric measurements, functional capacity and self-reported number of sick days, musculoskeletal symptoms, self-reported health, work ability, work productivity, physical capacity, kinesiophobia and physical functional status.

**Discussion:**

The results from this study will contribute to the knowledge about evidence-based interventions for prevention of sickness absence among health care workers.

**Trial registration:**

ClinicalTrials.gov: NCT01543984.

## Background

The primary cause of people staying on long-term sick leave is pain in the back and neck and other musculoskeletal disorders. For this reason musculoskeletal disorders were one of the Danish government's four priority health issues for 2010 [1]. A public health report from Denmark states that health care workers have relatively frequent complaints of musculoskeletal disorders [2]. Female health care workers in both 24-hour care centres and primary home care make up one of the seven occupations in Denmark with highest risk of long-term sick leave, incapacity benefit and early retirement [3].

Health care workers are at high risk of long-term sick leave as they have many physical work demands, involving patient handling and manual tasks and their work gives high peak heart rates of short duration. Their work is characterized by long periods of standing and walking as well as frequent awkward postures that are potentially harmful for the low back and shoulders [4]. Moreover, the demographic increase in age in the general population can lead to an increased need for and pressure on the nursing and hospital sector. This may result in a need for heightened efficiency placing increasing work pressure on employees in the health care sector. Any increase in the number of dependent elderly people may result in a parallel development of increased musculoskeletal stress among health care workers [3] as a consequence of greater stress both in the physical and the mental work environment.

A study of nursing personnel by Souza et al. [5] shows that the numbers reporting musculoskeletal symptoms in at least one body part during the past 6 months and the past 7 days were 80% and 50%, respectively. The nursing personnel in the study by Souza et al. most often reported symptoms in the low back, upper back and shoulders. Correspondingly, pain and discomfort that most often prevented them in completing activities were located in low back, upper back, wrists and hands. Musculoskeletal disorders are often recurrent, may result in chronic pain and may affect employees functional capacity and prohibit their work. It is important to preserve workers’ ability to perform their tasks at work, a conclusion sustained by results from Pohjonen [6] regarding the need to prevent the decline of working ability among home care workers. Alongside depression, pain-related work interruption and work-related pain had the largest total effect on the duration of work absence [7]. Therefore, focusing on pain is particularly important in interventions aiming at preserving or enhancing work ability in order to prevent work absence or reduce its duration [8].

Beside pain, low back disorders and previous sick leave are associated with a higher risk of future sick leave than any other cause [9]. The design of rehabilitation programmes for health care workers for the prevention of work disability should therefore focus on the employees previous history of pain related to the upper body, in addition to sick leave due to musculoskeletal disorders in back or upper body [9]. Activities for the prevention of work disability for health care workers will address not only the preservation or enhancement of work ability but also the potential to improve quality of work life [7].

It seems reasonable to focus on strategies for reducing physical loads on the home care worker, as well as finding a reasonable match between physical work load and the individual physical capacities [4,10]. An noticeable ongoing development and use of technical aids in care and hospital sector is likely to reduce musculoskeletal loads [3]. However, the strategy we pursue in the present study is to improve the physical capacity of the health care worker in order to prevent musculoskeletal disorders and preserve or enhance work ability. It is important to maintain physical capacity, especially, for workers with high physical work demands. Some studies have shown an association between high physical workloads and low physical capacity. The association between previous physical work loads and low muscle strength in the trunk and lower extremity and low aerobic power was especially evident among women with long-lasting high physical demands at work [10,11]. Pronk et al. [12] found that higher levels of physical activity for workers were related to maintained quality of work performed and overall job performance.

A systematic review of the effectiveness of community-based and workplace-based interventions to reduce musculoskeletal-related sickness absence and job loss concluded that no single intervention is more effective than another, regardless of whether they involve, for example exercises or education in behavioural changes. In addition, there was evidence that effort-intensive interventions are no more effective than more simple interventions [13].

In the present paper a study of 'Tailored Physical Activity’ (TPA) is conducted to investigate the effect on health care workers. The intervention takes the participants' pain history into consideration. Physical activity interventions involving exercises to strengthen muscles have been tested among various occupational groups to enhance their physical capacity and have proven to be effective in reducing pain and improving muscular strength [14,15]. Moreover, greater functional capacity, as measured by cardiorespiratory fitness, is related to increased quantity of work performed, and a higher level of cardiorespiratory fitness is related to a lesser effort exerted when performing certain work tasks [12]. Among job groups with sedentary work efficacy of physical activities have been shown for prevention of musculoskeletal disorders [16] and previous studies recommend to include both strength training and aerobic fitness training in preventive activities [17]. However, there is a lack of evidence on preventive activities among job groups with physical heavy work and activities for prevention of sickness absence and job [13]. This study will add knowledge on preventive activities for a specific job group with physical heavy work. It is a standardized intervention however individually tailored and conducted by health professionals.

It is expected that TPA enhances the health care workers' physical capacity and thereby reduces musculoskeletal loads. The larger physical resources for their work potentially will reduce pain and with it their days with sickness absence. Based on earlier registrations we expect that 50% of the health care workers have had one or more days off work due to musculoskeletal pain during the last 3 months. In continuation of this we expect that the TPA will show an effect so the proportion that reports at least one day on sick leave will be reduced to 15%.

Using a randomised controlled trial design, the aim of this study is to evaluate the efficacy of “Tailored Physical Activity” (TPA) versus a reference group (REF) in reducing the number of self-reported days with sick leave. The intervention in the study arm is carefully chosen on the basis of previous evidence-based studies that have shown to be effective in other occupational groups [15,18-23].

Outcome evaluations will be performed 3 months (immediately after the end of the TPA intervention) after baseline and 12 months (long-term) after baseline.

## Methods/Design

### Study design

**Figure 1:**
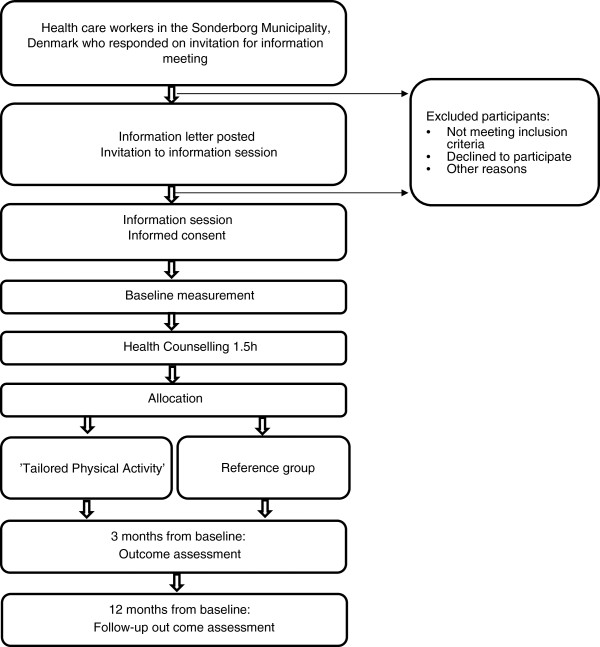
Flow diagram of the progress through the study.

This study is a parallel randomised single-blind controlled trial. It will evaluate the efficacy of TPA including general aerobic training and specific strength training versus REF on the participants self-reported number of days on sick-leave as illustrated in the flow diagram in Figure 1.

The study is conducted in the Sonderborg Municipality, Denmark. The trial duration is January 2012 to April 2014.

The study will utilize an allocation concealment process, ensuring that the group to which the participants are allocated, is not known before the participant are enrolled in the study.

To monitor the conduct of the study, a project steering group has been appointed. It consists of the participating scientists from the Institute of Sports Science and Clinical Biomechanics at University of Southern Denmark, the head of Senior Citizen and Health Department in the Sonderborg Municipality, the project manager from the Sonderborg Municipality, two department heads and three coordinators from the Health Care Centre.

The protocol is approved by The Regional Scientific Ethics Committee for Southern Denmark (project-ID S-20110040) and The Danish Data Protection Agency. The trial is registered in the ClinicalTrials.gov, number NCT01356784. All of the participants will give written informed consent before enrolment.

### Settings

The participants will be recruited from employees in the section 'Social and Health Affairs’ in the Sonderborg Municipality. Pre- and post-intervention tests and assessments, in addition to the interventions, will be performed at the Health Care Centre in Sonderborg.

### Study population

Eligible for the study will be health care workers employed by the Municipality of Sonderborg. Health care worker is a heterogeneous title and include participants working in the primary health care, e.g. nursing homes, home care or centre for substance abuse.

The inclusion criteria are: 1) health care workers performing manual work and 2) self-reported work related musculoskeletal pain in back or upper body.

Before invitation to an information meeting the eligible are interviewed to check if they meet the inclusion criteria. They are asked: 1) Are you performing manual work during a typical working day? and 2) Do you experience musculoskeletal pain related to your everyday work?

Excluded participants or eligible participants who do not want to participate will be registered in one of three categories, as recommended by the CONSORT statement: (1) Not meeting the inclusion criteria, (2) Declined to participate, or (3) Other reasons [24].

### Procedure for recruitment, randomization and allocation

Eligible participants who fulfill the inclusion criteria are invited to an information meeting. After the meeting, eligible participants willing to participate will sign a written informed consent for participation in the study.

The recruited participants are randomised in permuted blocks of 2 and 4 according to computer-generated random numbers, to participate in either TPA or REF.

To ensure concealment of the assigned intervention, a secretary in the administration of Social and Health Affairs in the Sonderborg Municipaltiy will obtain the opaque, sealed envelope containing the participant’s assigned intervention after the participants have received health guidance and just before the intervention is initiated. Neither the investigator nor the health personnel in the Health Care Centre has any other role in the sequence generation and subsequent allocation concealment.

### Interventions

Participants will be randomised to one of two arms. All randomised participants will receive health guidance for 1.5 hours from a trained supervisor. Additionally, the intervention group will be offered TPA. The intervention will start within one week from baseline measurements, health guidance and the randomization.

*Health guidance* is a 1.5-hour dialogue between the participant and health supervisor, based on the participant's lifestyle, motivation, resources and power to act. During the conversation the participant will have the opportunity to prepare a goal-oriented health plan identifying the means to achieve the changes that the participant wants and needs. The health supervisor will inspire and support the participants to take an active part in their own lives, such as increasing well-being in everyday life, physical activity and weight loss, as well as smoking cessation.

#### ***Tailored Physical Activity-group (TPA)***

This intervention group will receive TPA in addition to health guidance.

TPA sessions will be performed in teams of up to 10 participants and include a standardised combination of aerobic fitness and strength training for 50 minutes 3 times per week over 10 weeks supervised by a physiotherapist at the Health Care Centre in the Sonderborg Municipality. The participants will be referred to one of three standardised training programmes based on their primary region of musculoskeletal problems (neck and shoulder pain, arm and/or hand pain, lower back pain).

The three standardised training programmes all start with 5 minutes warm up during which the participants will gradually increase their heart rate (HR) followed by aerobic fitness training for 20 minutes, at intensities ranging from 50% with a progression up to 80% heart rate reserve. During the following weeks, training will be tailored to the participant’s current training status and pain problems [25].

For the warm up and the aerobic fitness training, the participants can choose between ergometer cycling, rowing, stepping or cross training. The choice is taken after consultation with the physiotherapist taking into consideration the participant’s current musculoskeletal troubles and general health. The relative workload will be estimated based on the known relationship between HR and oxygen uptake, i.e. relative workload = (working HR – resting HR) / (maximum HR – resting HR). Resting HR is set at 70 beats per minute and maximum HR is estimated at 208 – (0.7 × age) [26]. HR is monitored during each training session to ensure an optimal training intensity.

Participants with pain that is related to the upper body and the neck are referred to high-intensity strength training in modified programmes [15,18,19,22,23]. The programme for neck and shoulder pain contains five different dumbbell exercises; one-arm row, shoulder abduction, shoulder elevation, reverse flies and upright row. The participants with pain primarily located in the arm and/or hand region will participate in a programme with five different dumbbell exercises: front raise, shoulder abduction, reverse flies, shoulder elevation and wrist extension.

During the intervention period, the training load will progressively be increased from 15 repetitions maximum (~70% of maximal intensity) at the beginning of the training period to 8–12 repetitions maximum (~75–85% of maximal intensity) during the later phase. The strengthening exercises will be performed in a conventional manner using consecutive concentric and eccentric muscle contractions. Three of the five different exercises with three sets per exercise will be performed during each training session in an alternating manner, with shoulder elevation being the only exercise that is performed during each session [18].

Participants with symptoms in the lower back will be referred to specific strength training similar to the exercises for the upper body, in addition to coordination exercises for the lower spine. The exercises are standardised and composed of exercises activating the rectus abdominis, erector spinae and oblique externus muscles for more than 60% of their maximal voluntary contraction [20,21]. The rate of progression of all the exercises will be controlled and depend on strength gains.

Only physiotherapists educated in accordance with the manuals for the training concepts will take part in the project to ensure standardised guidance and supervision. The physiotherapists will be encouraged to use their professional judgment to calibrate each participant’s programme based on the response of their musculoskeletal condition to the physical demands of the programme and also use their professional judgment according to optimize the programme to reduce the participants sickness absence related to musculoskeletal pain. The training activity will be recorded in a diary by the end of each session.

#### ***Reference-group (REF)***

The REF group will receive health guidance only.

### Outcome measures

Measurements will take place at baseline and at the end of the intervention, approximately after 3 months. A secondary follow-up measurement will be performed 12 months after baseline to examine long-term effects. Baseline demographic characteristics of participants will also be recorded.

The *primary endpoint* for efficacy will be participants’ self-reported sickness absence because of musculoskeletal troubles. It will be evaluated with a modified question from the Nordic Musculoskeletal Questionnaire “How many days in total have you been on sick leave because of musculoskeletal trouble (such as ache, pain, discomfort) during the last 3 months?” (0 days, 1–7 days, 8–30 days, >30 days) [27].

Secondary endpoints will include objective measures of anthropometry, hand-grip strength and aerobic capacity. In addition, we will evaluate self-reported measures of musculoskeletal symptoms, self-reported health, work ability, work productivity, physical capacity, kinesiophobia, physical functional status, interpersonal problems and mental disorders, via questionnaires.

Objective measurements will be performed by trained physiotherapists. Hand-grip strength will be measured in kilograms with a digital hand-held dynamometer. Participants will be instructed to sit upright on a chair with the safety strap around their wrist, with their arm at right angles and their elbow by the side of their body. Wrist extension only up to 30° will be allowed. The participants will be strongly encouraged to squeeze with maximum effort. Three trials will be recorded and an extra trial will be conducted if force is changed more than three kilograms compared with the previous attempts [28].

Aerobic capacity will be estimated with the Aastrand-Rhyming Test, which is a submaximal cycle ergometer aerobic fitness test. The participants will cycle 60 rpm at a work load set at a level referenced to the sex and condition of the subject. The participant’s HR is measured during the exercise and the test will be terminated when the subject reaches a steady state HR of between 120 and 160 beats/min, with a change of less than 5 beats between two consecutive minutes. Aerobic capacity will be estimated based on Aastrands nomogram, using the participant’s work-load and HR during testing [29]. Finally, the result will be adjusted for age and gender, normalised to body weight.

Musculoskeletal symptoms in the shoulder, elbow, hand, neck, upper back and lower back will be evaluated with a modified version of the Nordic Musculoskeletal Questionnaire. The questions used are “Have you, at any time during the last 3 months had trouble (such as ache, pain, discomfort) in [body part]?” (yes/no), “How many days have you had trouble (such as ache, pain, discomfort) in [body part] during the last 3 months?” (0 days, 1–7 days, 8–30 days, >30 days but not every day, every day), “How many days in total have you been on sick leave because of trouble (such as ache, pain, discomfort) in [body part] during the last 3 months?” (0 days, 1–7 days, 8–30 days, >30 days), “Because of trouble (such as ache, pain, discomfort) in [body part] have you been examined or treated by a doctor, chiropractor or physiotherapist or the like during the last 3 months (yes/no), “Have you had trouble (such as ache, pain, discomfort) in [body part] during the last 7 days?” (yes/no). Illustrations from the Nordic Questionnaire define the respective body regions of the neck, right shoulder, left shoulder, upper spine, lower spine, right elbow, left elbow, right hand and left hand [27].

Self-reported health and health-related quality of life will be measured using the SF-36 Health Survey, a standardised questionnaire investigating eight health concepts: physical functioning, role limitations because of physical functioning, bodily pain, general health, vitality, social functioning, role limitation because of emotional problems and mental health. Answers are recorded using a Likert scale [30,31].

Work ability will be assessed by the single-item measure that was originally part of the widely used Workability Index. However, recent studies have shown that the single item question is a reliable and easy tool with validity comparable with the full index [32]. The question used is “Imagine that your work ability is worth 10 points when it is at its best. How many points would you give your present work ability?” A numerical rating scale was used where 0 represents “not able to work” and 10 represents “the highest work ability” [33].

Work productivity will be assessed with two questions modified from Work Performance Questionnaire [34]: “During the past month, how much did health problems affect …” 1)”…your quality of work while you were working?” and 2) “…your productivity while you were working?” Answers are recorded using a Likert Scale where 1 represents “A high extent and 5 represents “Not at all”.

Kinesiophobia are dysfunctional beliefs about physical activities that will be assessed using the Tampa Scale for Kinesiophobia. It is a 17-item questionnaire to assess fear of (re)injury due to movement, because avoidance behavior can be one mechanism in sustaining chronic pain disability. Each item is provided with a 4-point Likert scale with scores ranging from “strongly agree” to “strongly disagree” [35-37].

Perceived disability in terms of self-reported activity limitation for the primary region of pain will be measured by the Neck Disability Index (NDI), Disabilities of the Arm, Shoulder or Hand (DASH) or Roland Morris Disability Questionnaire (RMQ).

The NDI is a 10-item questionnaire designed to measure disability in activities of daily living due to neck pain. Each item has 6 response options ranging from no pain and no functional limitation to worst pain and maximal limitation [38,39].

The DASH is a 30-item questionnaire with five response options for each item. It is designed to measure physical function and symptoms for musculoskeletal disorders of the upper limb [40,41].

The RMQ is a 23-item questionnaire that assesses the degree of function and disability due to low back pain and/or sciatica. Each item is scaled yes/no. 'No’ corresponds to no disability and 'yes’ corresponds to self-rated disability on each item [42].

Self-assessed physical fitness will be evaluated using a questionnaire based on Stroyer et al. but modified from a VAS-scale to a Likert scale [43]. It consists of five items with illustrations of five situations reflecting aerobic fitness, muscle strength, endurance, flexibility and balance. The participants will be asked “How would you rate the following components of physical fitness compared with others of your own age and sex”?. A Likert scale will be used where 1 represents “poor” or “weak” and 10 represents “good” or “strong”.

### Blinding

Health care professionals and participants will be aware of the allocation arm but blinded to the results of any former assessment. Health care professionals who are outcome assessors will be blinded to participants’ allocation and former assessment.

### Sample size

The participants self-reported sickness absence will be analysed as a dichotomous measure indicating whether the participant has had no sickness absence (0 days) because of musculoskeletal troubles or has had sickness absence during the last three months (1–7 days, 8–30 days, >30 days). Assuming that 15% or less of the health care workers in the TPA group and 50% of the participants in the REF group report they have had sickness absence within the last three months, a sample size of 22 individuals in each group will be required to achieve greater than 80% statistical power (one-sided, alpha = 0.05).

### Statistical analysis

The primary analysis for this study will be conducted according to the intention-to-treat principle where the study participants will be analyzed according to what arm they were randomised, independent of their degree of participation. Univariate frequencies or means with 95% confidence intervals will be calculated to describe the demographics and baseline characteristics of the two arms. Our primary analysis will compare the proportion of self-reported sickness absence from work due to musculoskeletal complaint at three months follow up, between the two arms using a chi-square test. Study participant characteristics that vary between the two arms at baseline will be included in a multivariable logistic regression model to test the primary research question after controlling for the potential confounders.

Continuous secondary outcomes will be analyzed using linear mixed models. Significant differences between groups in baseline characteristics will be included in the model.

## Discussion

This study make a further contribution to the evidence base of initiatives for improving physical capacity for heavy work such as that performed by the health care workers. Strategies in the workplace aimed at enhancing physical capacity and/or the ability to cope with musculoskeletal pain have been successfully tested [15,18-23] but mainly among sedentary workers. On the other hand, a review of the effectiveness of physical activity programmes carried out at worksites has shown limited evidence on reducing absence [44]. A study of Brox et al. [45] found that fitness training did not reduce sickness absence. However, this conclusion can probably be explained by a low frequency and intensity of training. As stated in a review of dose–response relation between physical activity and sick leave, there is no positive relationship between moderate physical activity and sick leave, only vigorous physical activity having a positive effect [46]. Therefore, it seems crucial to the outcome, defined as reduce sick leave, that physical activity interventions have high frequency and intensity, with a threshold value of three times a week [46]. The interventions in the present study encompass a combined strategy of aerobic fitness training and strength training. This strategy has not been studied for efficacy with respect to sickness absence but has been applied as 'return to work’ intervention [47]. Both these interventions are high intensity interventions, while taking into consideration the participant’s current musculoskeletal troubles and general health.

Publishing the design of a study before the study is performed and the results obtained has several advantages. It allows the design to be finalised without being influenced by the outcomes. This can assist in preventing bias as deviations from the original design can be identified.

The present research design is composed as an 'add-on’ design. While both groups receive health guidance, the intervention group receives additional TPA. The reason why all participants receive health guidance is that we consider it unethical not to offer some form of treatment, i.e. randomizing the control group to a waiting list.

## Competing interests

The authors declare that they have no competing interests.

## Authors’ contributions

LNA, LGH and KS initially designed the study. All authors contributed to developing the protocols and intervention materials. LNA and KS drafted the manuscript and all authors were involved in revising it for intellectual content and have given final approval of the version to be published. All authors read and approved the final manuscript.

## Pre-publication history

The pre-publication history for this paper can be accessed here:

http://www.biomedcentral.com/1471-2458/13/917/prepub
